# Effects of carbon ion beam alone or in combination with cisplatin on malignant mesothelioma cells *in vitro*

**DOI:** 10.18632/oncotarget.23756

**Published:** 2017-12-29

**Authors:** Sei Sai, Masao Suzuki, Eun Ho Kim, Mitsuhiro Hayashi, Guillaume Vares, Naoyoshi Yamamoto, Tadaaki Miyamoto

**Affiliations:** ^1^ Department of Basic Medical Sciences for Radiation Damages, National Institute of Radiological Sciences, National Institutes for Quantum and Radiological Science and Technology, Chiba, Japan; ^2^ Division of Applied Radiation Bioscience, Korea Institute of Radiological and Medical Sciences, Gongneung-dong, Nowon-Gu, Seoul, South Korea; ^3^ Breast Center, Dokkyo Medical University Hospital, Mibu-machi, Shimotsuga-gun, Tochigi, Japan; ^4^ Okinawa Institute of Science and Technology (OIST), Advanced Medical Instrumentation Unit, Onna-son, Okinawa, Japan; ^5^ Hospital of the National Institute of Radiological Sciences, National Institutes for Quantum and Radiological Sciences and Technology, Chiba, Japan; ^6^ Chiba Foundation for Health Promotion and Disease Prevention, Chiba, Japan

**Keywords:** heavy-ion radiation, mesothelioma, cisplatin

## Abstract

Malignant mesothelioma (MM) is extremely aggressive and a typical refractory cancer. In this study we investigated how effective on killing MM cells by carbon ion beam alone or in combination with cisplatin (CDDP) *in vitro*. Carbon ion beam (at the center of SOBP with 50 keV/µm of average LET) dose-independently suppressed MM cells MESO-1 and H226 cell viability and in combination with CDDP (25 μM) significantly enhanced its action. Relative biological effectiveness (RBE) values at 73 keV/μm and 13 keV/μm portion of carbon ion beam was estimated as 2.82-2.93 and 1.19-1.22 at D10 level relative to X-ray, respectively by using colony formation assay. Quantitative real time PCR analysis showed that expression of apoptosis-related BAX and autophagy-related Beclin1 and ATG7 was significantly enhanced by carbon ion beam alone or in combination with CDDP. Apoptosis analysis showed that caspase 3/7 activity and the percentage of apoptotic cells was dose-dependently increased after carbon ion beam and it was further increased when combined with CDDP. Spheroid formation ability of cancer stem like CD44+/CD26+ cells was significantly inhibited by carbon ion beam combined with CDDP. Besides, carbon ion beam combined with cisplatin significantly inhibited cell cycle progression (sub-G1 arrest) and induced more large number of γH2AX foci. In conclusion, carbon ion beam combined with CDDP has superior potential to kill MM cells including CSCs with enhanced apoptosis.

## INTRODUCTION

Malignant mesothelioma (MM) has a long latency period and usually detected at the disease has already reached the advanced stages. Therefore the prognosis for patients with MM is often very poor; the 1-year survival rate for MM patients is about 40% and the 5-year survival rate is approximately 10% [1, 2].

Treatment using charged carbon ion beams is an emerging and promising form of radiotherapy that can target deeply located and radioresistant tumors, because of the high energy released by the “spread out bragg peak (SOBP) [3, 4]. Carbon ion beams have several advantages over conventional radiation, such as low dependence on cell cycle and oxygenation, and they can induce complex DNA damage compared to [5, 6]. In the past two decades, 11,000 cancer patients have been treated by carbon ion radiotherapy using HIMAC (Heavy Ion Medical Accelerator in Chiba), and the outcomes have been encouraging [7, 8]. However, some typical refractory cancer like MM, we still have not started clinical trials yet. There are also lack of basic biological studies about effects of heavy ion beams on MM cells.

Accumulating evidence indicates that tumors contain a small population of cancer stem cells (CSCs) that are possess characteristics of self-renewal and tumorigenic properties. Currently, CSCs are mainly identified using s cell surface markers that are specific for each tumor type. It has been reported that expression of cell membrane markers such as CD24, CD44, and CD26 is indicative of malignant mesothelioma (MM) CSCs [9, 10]. These CSCs are closely related with chemo-radioresistance, tumor relapse and metastasis [11, 12]. The efficient eradication of these CSCs is therefore the key in improving cancer curability [13, 14].

Recently, we have reported that carbon ion beam has a marked effect on colon and pancreatic CSCs [15, 16], and also shown that carbon ion beam combined with DNA damaging drugs has more power to kill those of radioresistant CSCs [17, 18]. Considering the fact that cisplatin (CDDP) is a highly effective chemotherapeutic agent for mesothelioma [19, 20], in the present study, we explored the mechanism through which carbon ion beams kill MM cells when used alone or induce DNA damage and alter the expression of apoptotic and autophagy-related genes when used in combination with cisplatin compared with photon beams. To our knowledge, this is the first study to report the effectiveness of carbon ion beams alone or in combination with cisplatin in targeting MM CSCs *in vitro*. This study may provide information for the development of new treatment strategies for this refractory cancer.

## RESULTS

### Viability of MESO1 and H226 cells after treatment with carbon ion beam irradiation alone or in combination with cisplatin

**Figure 1:**
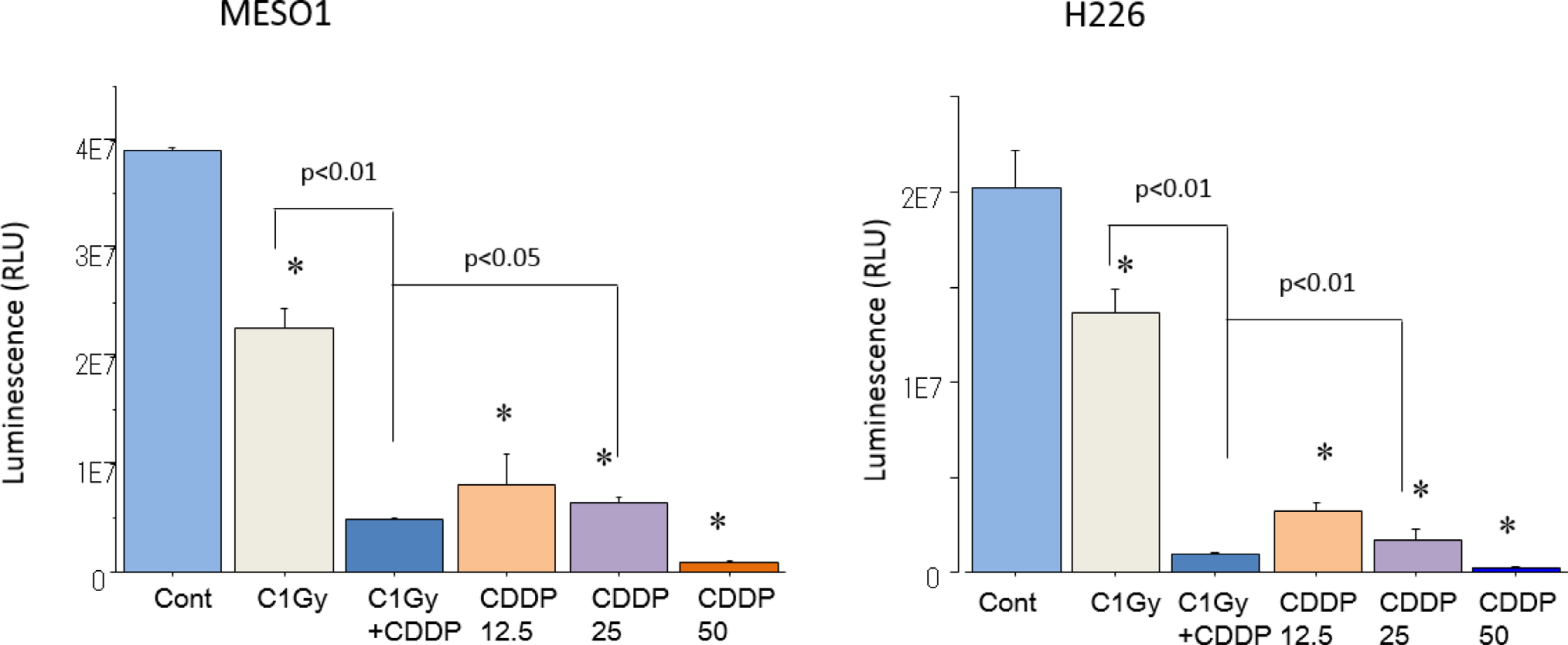
Cell viability analysis using CellTiter-Glo luminescent cell viability assay MESO1 and H226 cell viability was showed 7 days after carbon ion beam alone, 3 different concentration of cisplatin (CDDP, 12.5, 25, 50 µM ) alone or carbon ion beam in combination with 25 µM of cisplatin (CDDP). ^*^p < 0.01, compared to control.

Cisplatin is known to be highly effective in killing MM cells, and we hypothesized that when combined with heavy-ion irradiation, its cytotoxic effects could be enhanced. In order to clarify the radiosensitization effects of CDDP, the viability of MESO1 and H226 cells was estimated using CellTiter-Glo luminescent cell viability assay seven days after carbon ion beam irradiation (at the center of SOBP with 50 keV/µm of average linear energy transfer, or LET) alone or in combination with CDDP at three different concentrations (12.5, 25, 50 µM), as well as after X-ray irradiation alone, or in combination with CDDP (25 µM). Both MESO1 and H226 Cell viability was decreased by carbon ion beam alone and it was predominantly decreased after in combination with 25 µM CDDP. Cell viability was decreased by CDDP in a concentration-dependent manner (Figure 1).

### Surviving fraction of MESO1 and H226 cells after carbon ion beam alone or in combination with cisplatin

**Figure 2:**
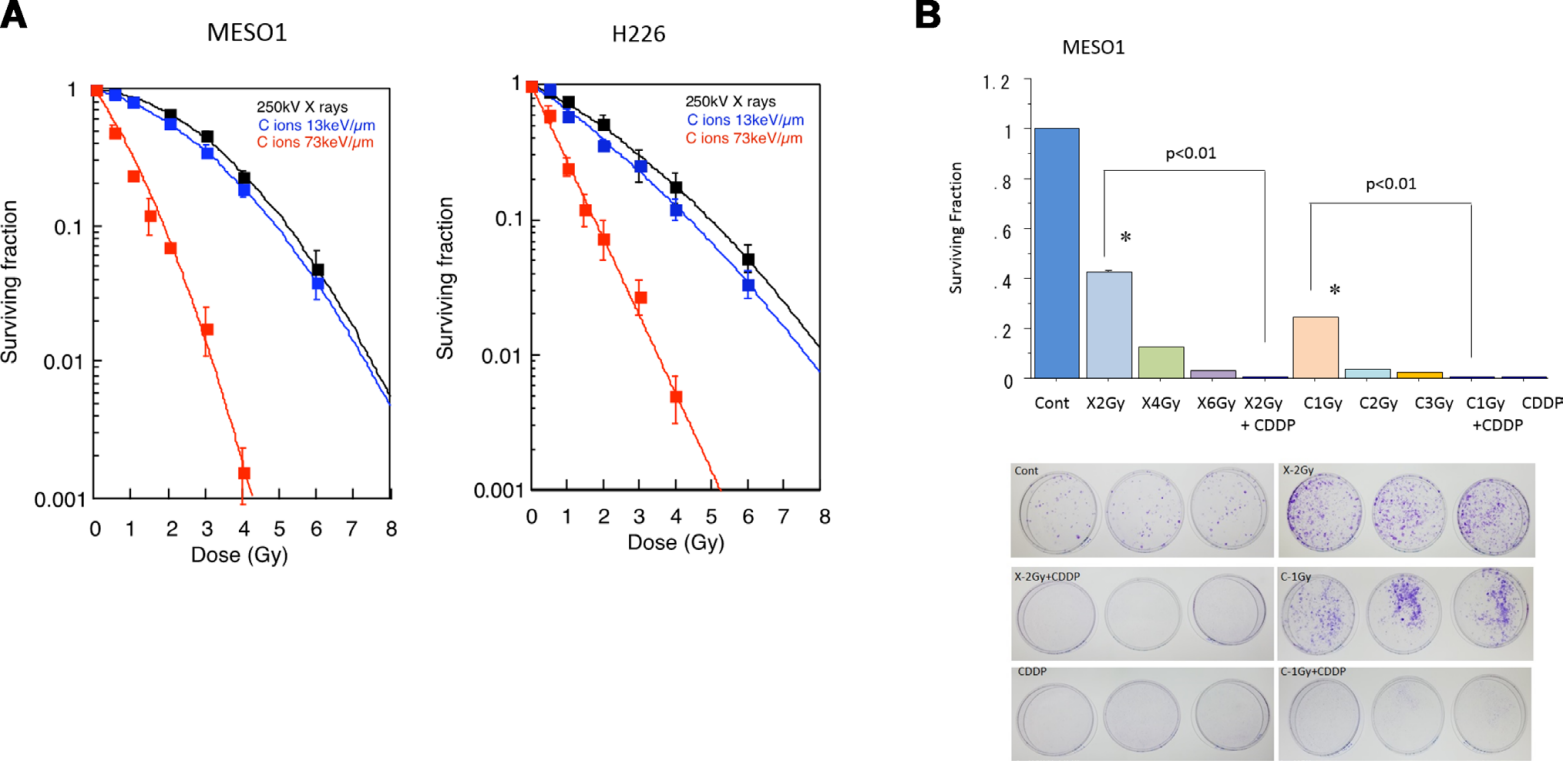
(**A**) Surviving fraction of MESO1 and H226 cells after carbon ion beam (the dose averaged LET values of 13 and 73 keV/μm at SOBP) or X-ray irradiation. (**B**) Surviving fraction of MESO1 after carbon ion beam (the 6-cm center of SOBP with averaged LET of 50 keV/μm) alone or in combination with 25 µM of CDDP. The cells plated immediately after carbon ion beam or X-ray irradiation. CDDP was added to the medium and continuously treated for 48 h and then replaced with new medium. The graphs show the mean and standard error calculated from three independent experiments. All experiments were performed in triplicate (*n* = 3).

To clarify cell killing effects of carbon ion beam with different LET, the dose averaged LET values of 13 and 73 keV/μm at SOBP were used. The H226 cells were irradiated with X-ray or carbon ion beams and their surviving fraction was estimated by colony assay. The survival of H226 cells after irradiation with carbon ion beam or X-rays decreased exponentially in a dose-dependent manner. As shown in Figure 2A, the clonogenic survival of MESO1 and H226 cells significantly decreased after exposure to X-rays or carbon ion beams (13 and 70 keV/μm, respectively). The average doses to reduce MESO1 (H226) cell survival to 10% were estimated to be 5.04 (5.44) Gy for X-rays, and 4.24 (4.46) Gy and 1.72 (1.93) Gy for carbon ion beams at 13 and 70 keV/μm carbon ion beams, respectively (Table 1). Accordingly, the relative biological effectiveness (RBE) values of carbon ion beams at 13 and 73 keV/μm relative to X-ray were calculated as 1.19 (1.22) and 2.93 (2.82), respectively (Table 1). In addition, we examined surviving fraction of MESO1 cells after carbon ion beam (at the center of SOBP with 50 keV/µm of average LET) alone or in combination with 25 µM CDDP. As shown in Figure 2B, the cell surviving fraction was dose-dependently decreased by either X-ray or carbon ion beam irradiation and combination with CDDP remarkably decreased the survival. Treatment with CDDP extremely inhibited colony formation even with relatively low concntration (12.5 µM). We also investigate surviving fraction of H226 cells after carbon ion beam (at the center of SOBP) alone or in combination with CDDP, and obtained same results as MESO1 cells (data not shown).

**Table 1:** RBE values at D10 level for MESO1 and H226 cells after carbon ion beam or X-ray irradiation

Cells	X-ray 200kV	C-ion (13 keV/μm)	RBE	C-ion (73 keV/μm)	RBE
MESO1	5.04 Gy	4.24 Gy	1.19	1.72 Gy	2.93
H226	5.44 Gy	4.46 Gy	1.22	1.93 Gy	2.82

### Confirmation of CSC-like characteristics of CD44+/CD26+ cells derived from MESO1 and H226 cells

**Figure 3:**
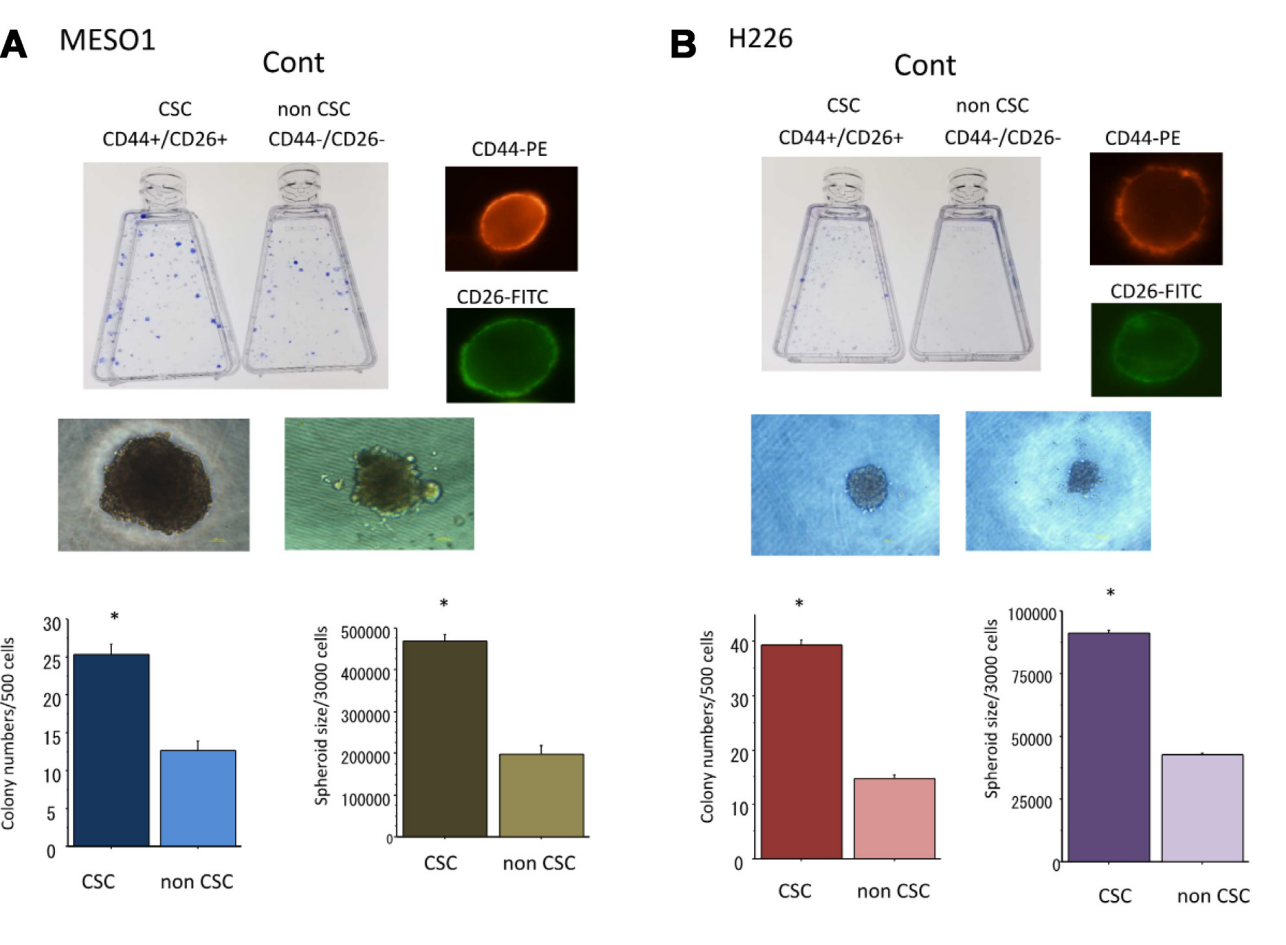
**(A)** Colony and spheroid formation of cancer stem-like cells (CSCs) (CD44+/CD26+) and non-CSCs (CD44–/CD26–) delivered from MESO1 cells. The cells were cultured for 1–2 weeks for colony and spheroid formation ability analyses. (**B**) Colony, spheroid formation and tumorigenicity of cancer stem-like cells (CSCs) (CD44+/CD26+) and non-CSCs (CD44–/CD26–) delivered from H226 cells. The cells were cultured for 1–2 weeks for colony and spheroid formation ability analyses. Representative photos of CSCs are also displayed. ^*^p < 0.01, compared to colony or sphere formed from non-CSCs. All experiments were performed in triplicate (*n* = 3).

To confirm the CSC-like characteristics of CD44+/CD26+ cells, we performed assays to evaluate colony and spheroid formation capability. We found that CD44+/CD26+ cells had greater capability of colony and spheroid formation than CD44–/CD26– cells did (Figure 3). Briefly, when the same number of cells (500) were plated in a dish, CD44+/CD26+ cells derived from MESO1 and H226 formed 25 + 3 and 40 + 3 colonies. In comparison, CD44–/CD26– cells formed only 12 + 2 and 14 + 2 colonies (*p* < 0.01), respectively. These data indicated that CD44+/CD26+ cells had great colony formation capability than did CD44–/CD26– cells (Figure 3A). When the same number of cells (3000) were cultured in 96-well round-bottomed Sumilon Celltight spheroid plates (Sumilon, Sumitomo Bakelite Co., Tokyo, Japan) for one week, the spheroids formed from CD44+/CD26+ cells were not only remarkably higher in number but also larger than those formed from CD44–/CD26– cells (*p* < 0.01) (Figure 3B).

### Changes in proportion of CD44+/CD26+ cells following carbon-ion irradiation alone or in combination with cisplatin

**Figure 4:**
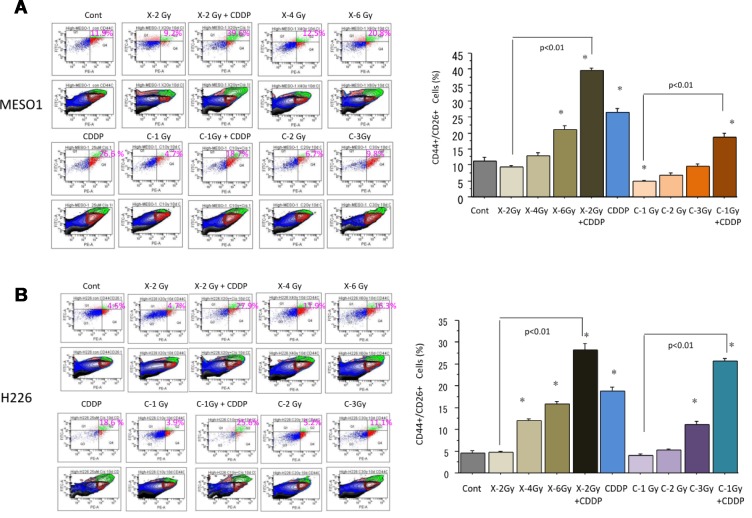
(**A**) Percentage changes of CD44+/CD26+ cells by FACS analysis 10 days after carbon ion beam or X-ray irradiation alone or in combination with 25 µM of cisplatin (CDDP) in MESO1 cells. CDDP was added 1 h prior to irradiation and treated for 10 days. (**B**) Percentage changes of CD44+/CD26+ cells by FACS analysis 10 days after carbon ion beam or X-ray irradiation alone or in combination with 25 µM of CDDP in H226 cells. CDDP was added prior to irradiation and treated for 10 days. ^*^p < 0.01 compared to non-CSCs. All experiments were performed in triplicate (*n* = 3).

To investigate the changes in the proportion of CSC-like CD44+/CD26+ cells among H226 and MESO1 cells ten days after carbon ion irradiation (at the center of SOBP with 50 keV/µm of average LET), and X-ray irradiation alone or in combination with cisplatin (25 µM), fluorescence-activated cell sorting (FACS) analysis was performed. We found that the percentage of CD44+/CD26+ cells among MESO1 cells was increased after X-ray irradiation in a dose-dependent manner, whereas no such changes was induced by carbon ion irradiation (Figure 4A). As shown in Figure 4B, X-ray irradiation combined with cisplatin predominantly enhanced the proportion of CD44+/CD26+ cells compared to that of carbon ion irradiation combined with CDDP. We found that the percentage changes in CD44+/CD26+ cells among H226 cells after irradiation with carbon ion beams, X-ray alone or in combination with CDDP was similar to that observed in MESO1 cells.

### Spheroid formation capability of CD44+/CD26+ and CD44–/CD26– cells derived from H226 and MESO1 cells after carbon-ion irradiation or X-ray irradiation alone or in combination with cisplatin

**Figure 5:**
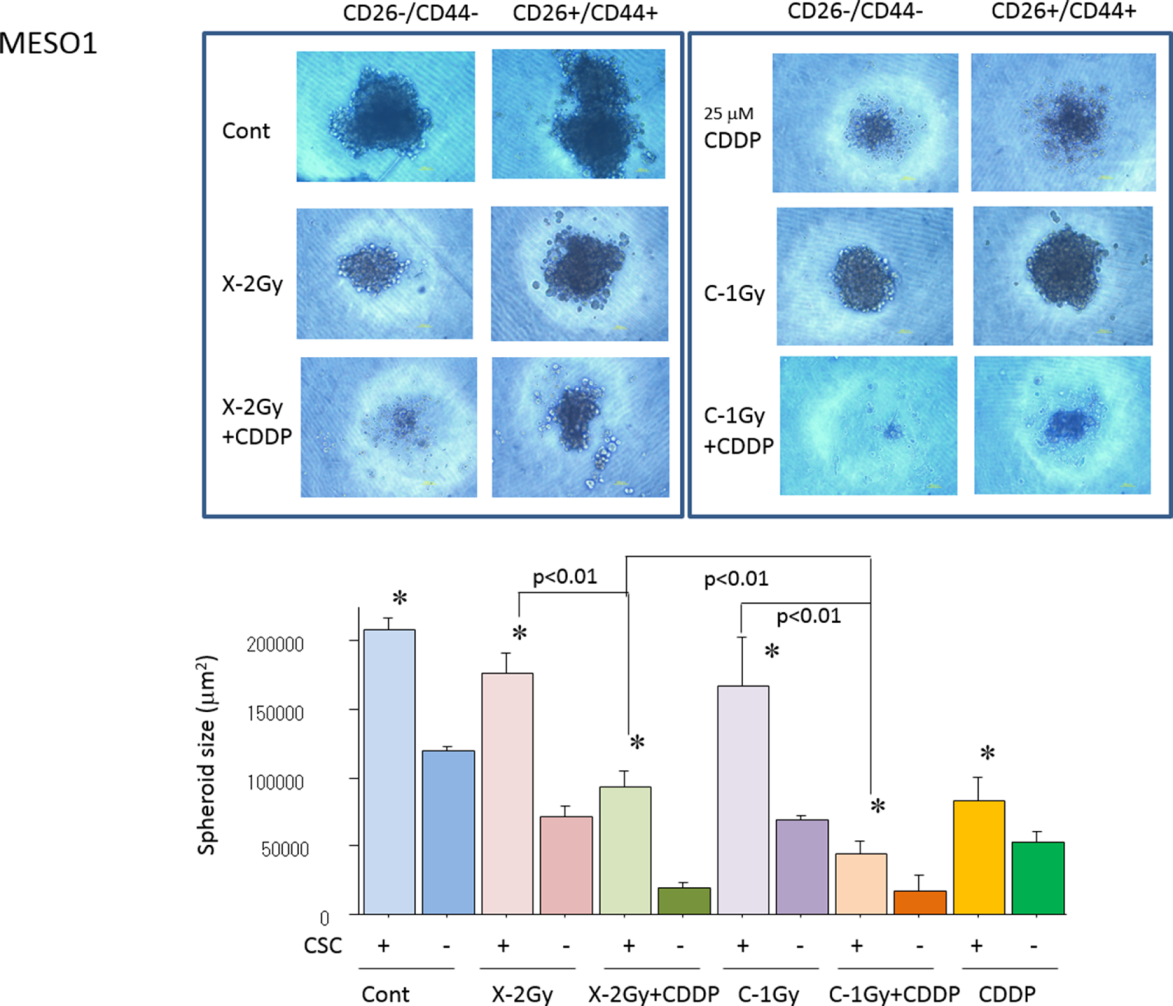
Quantification of spheroid formation of (CD44+/CD26+) and non-CSCs (CD44–/CD26–) after X-ray, a carbon ion beam alone or in combination with 25 µM of cisplatin (CDDP) CDDP was added prior to irradiation and treated for 7 days. Representative photos and quantification of spheroid size formed from MESO1 delivered CSCs (CD44+/CD26+) and non-CSCs (CD44–/CD26–) after X-ray, a carbon ion beam alone or in combination with 25 µM of CDDP. The spheroid formation was observed 7 days after X-ray, a carbon ion beam alone or in combination with CDDP. The graphs show the mean and standard error calculated from three independent experiments. ^*^p < 0.01, compared to non-CSCs. All experiments were performed in triplicate (*n* = 3).

To examine the effects of cisplatin on radiosensitization to X-rays and carbon ion beams, we performed spheroid formation capability assays on CD44+/CD26+ cells and CD44–/CD26– cells after X-ray irradiation or carbon ion irradiation (at the center of SOBP with 50 keV/µm of average LET) alone or in combination with cisplatin. As shown in Figure 5, the spheroid formation capability of CD44+/CD26+ cells derived from MESO1 after carbon ion irradiation alone but not X-ray irradiation remarkably suppressed spheroid size which was further decreased when carbon ion irradiation was combined with cisplatin.

### Expression changes of apoptosis- and autophagy-related genes in CSCs after carbon-ion beam alone or in combination with cisplatin by real time RT PCR analysis

**Figure 6:**
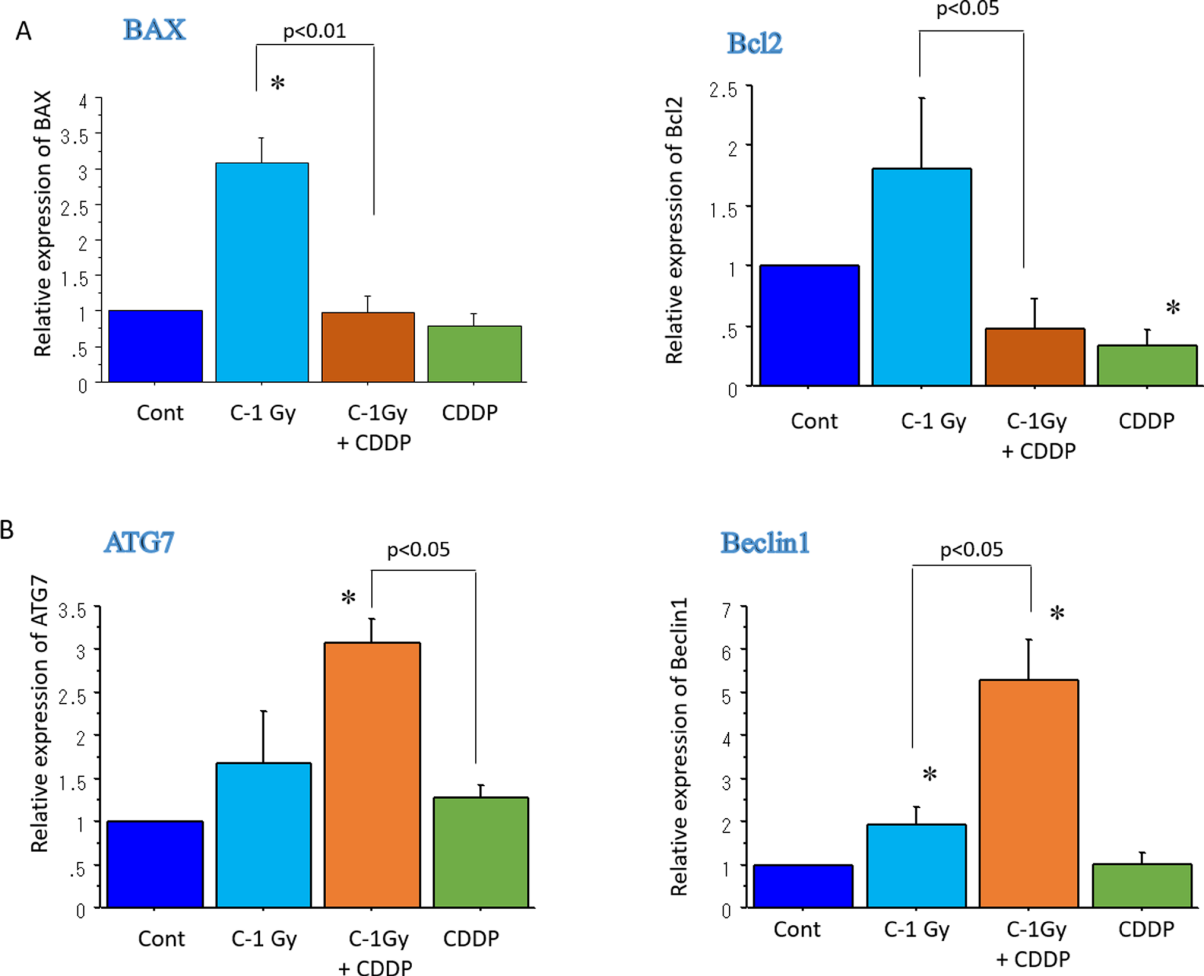
Real time RT-PCR analysis of expression changes of apoptosis- (**A**) and autophagy-related genes (**B**) 96 h after carbon ion beam alone or in combination with CDDP (25 μM) in CSCs derived from MESO1 cells. ^*^p < 0.05, compared to control. All experiments were performed in triplicate (*n* = 3).

To quantitatively examine apoptosis- and autophagy-related gene expression changes in radioresistant CSCs (CD44+/CD26+) delivered from MESO-1 cells, real time RT PCR analysis was performed according to the manufacture’s protocol. The data shows that treatment with a carbon ion beams (at the center of SOBP with 50 keV/µm of average LET) alone or combined with constant treatment with 25 μM of cisplatin for four days significantly increased the expressions of apoptosis-related BAX expression and decreased Bcl2 expression (*p* < 0.01). In addition, carbon ion beam combined with cisplatin significantly increased the expression of autophagy-related genes Beclin1 and ATG7 expression (*p* < 0.01) compared to that observed with carbon ion beams, or cisplatin alone (Figure 6).

### Apoptosis analyses of MESO1 cells after carbon-ion beam alone or in combination with cisplatin

**Figure 7:**
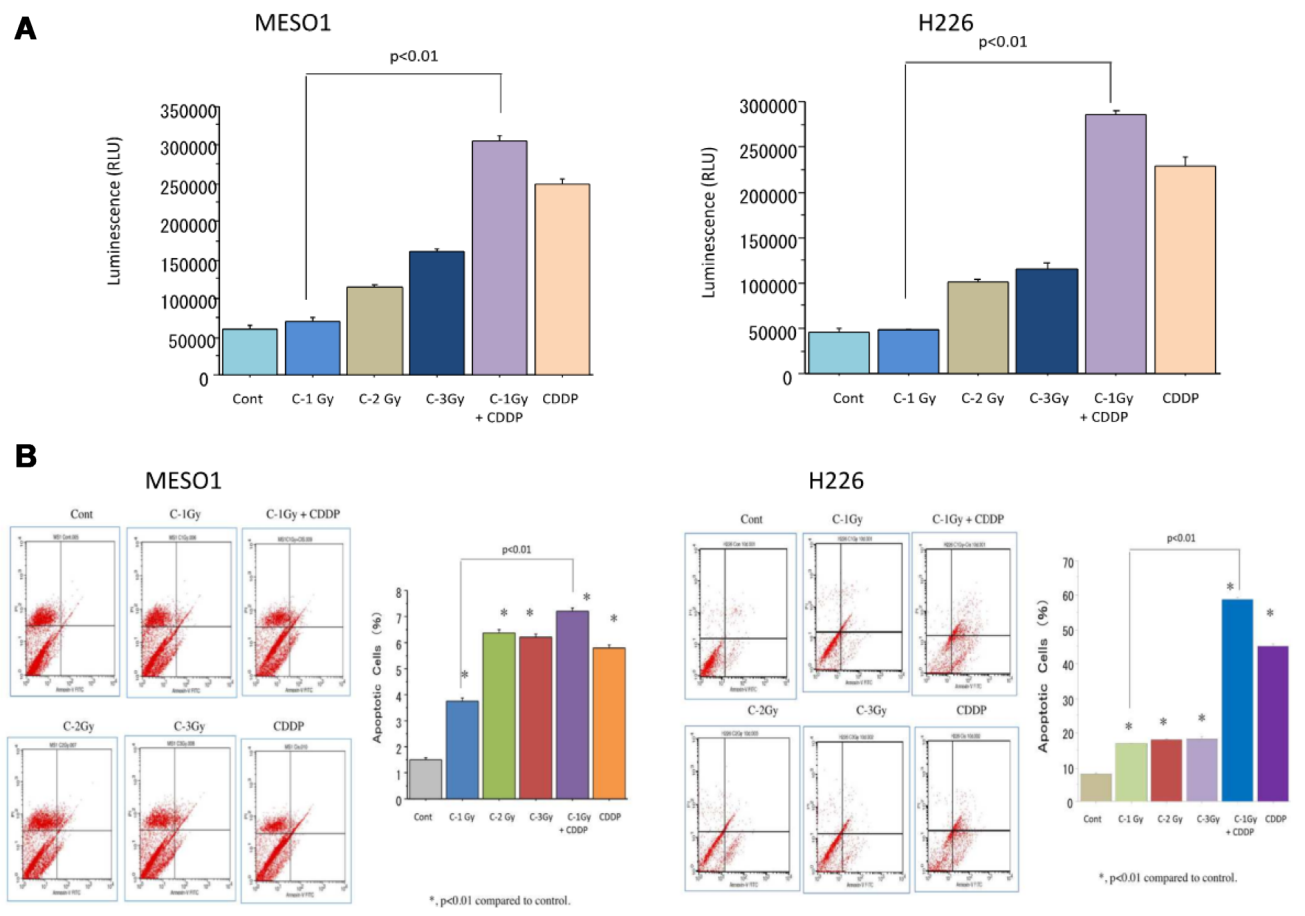
(**A**) Caspase 3/7 activity analysis of MESO1 and H226 cells 72 h after a carbon ion beam, alone or in combination with 25 µM of cisplatin (CDDP) by Caspase-Glo™ 3/7 Assay kit. (**B**) Apoptosis analysis of MESO1 and H226 cells 10 days after a carbon ion beam alone or in combination with 25 µM of cisplatin (CDDP) by FITC Annexin-V-PI detection kit. ^*^p < 0.01, compared to control. All experiments were performed in triplicate (*n* = 3).

Apoptosis is considered to be one of the main cell death mechanisms following exposure to irradiation. To examine the apoptosis induction after carbon ion beam (at the center of SOBP with 50 keV/µm of average LET) alone or in combination with 25 µM of cisplatin (CDDP), we analyzed the apoptosis using caspase 3/7 activity assay by Caspase-Glo™ 3/7 Assay kit and Annexin V-FITC Apoptosis Detection Kits. The data showed that carbon ion beam alone dose-dependently increased caspase 3/7 activity, and it was further enhanced after in combination with CDDP (Figure 7A). FACS analysis using Annexin V-FITC Apoptosis Detection Kits indicated that carbon ion irradiation alone at 1 Gy significantly increased apoptosis. When combined with cisplatin, it produced greater enhancement of apoptosis than carbon ion beams, or cisplatin alone (Figure 7B).

### Cell cycle analyses of MESO1 cells after carbon-ion beam alone or in combination with cisplatin

**Figure 8:**
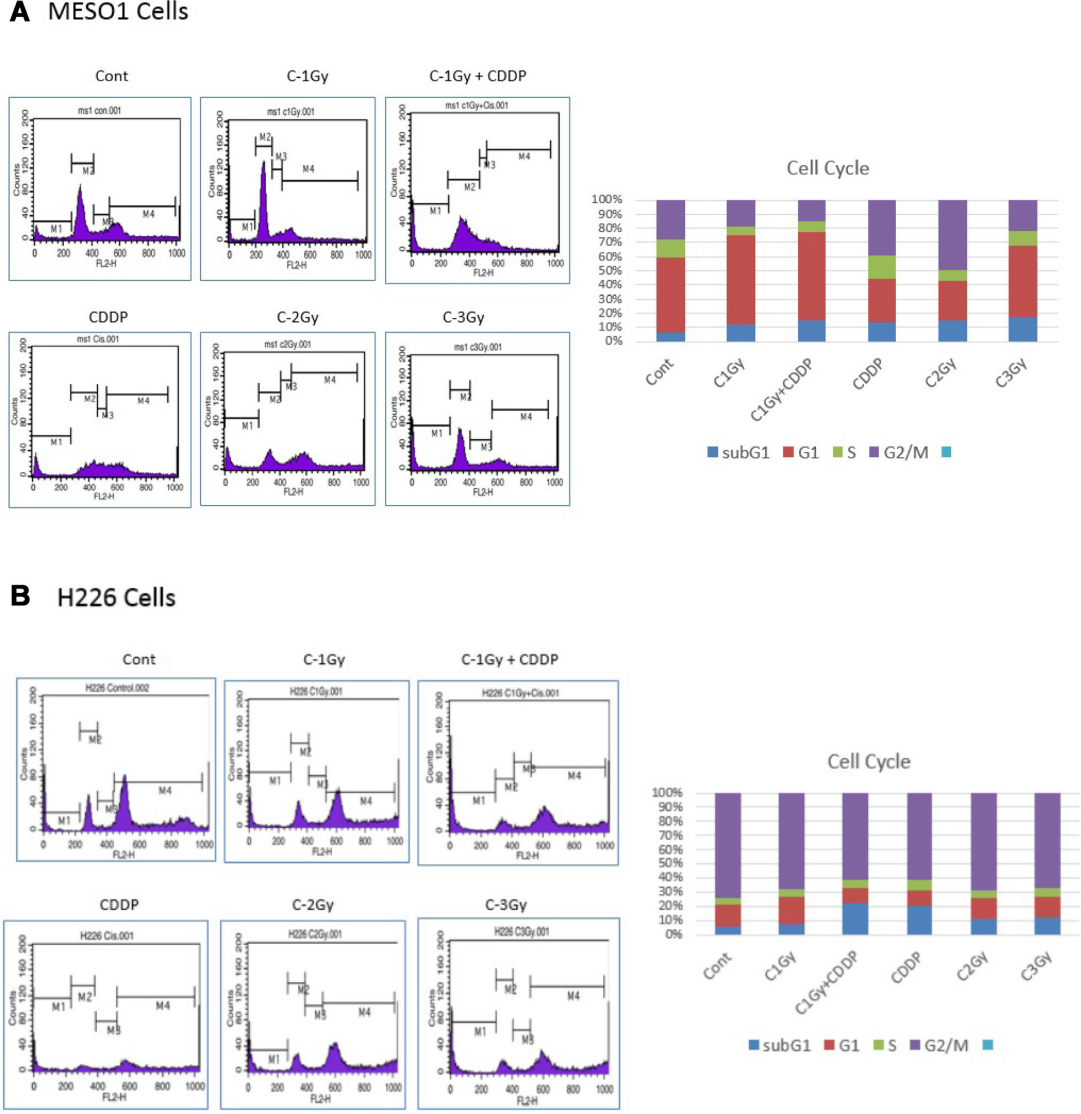
Cell cycle analyses of MESO1 (**A**) and H226 (**B**) cells 4 days after a carbon ion beam alone or in combination with 25 μM of cisplatin (CDDP). CDDP was added prior to irradiation and treated for 4 days, and the cell cycle distribution (sub G1, G1, S and G2/M phase) was measured by flow cytometry. Carbon ion beam combined with CDDP significantly inhibited cell cycle progression (sub-G1 arrest) and induced cell death (apoptosis/necrosis). Three separate experiments were conducted, and representative results are shown. Averages of the three separate experiments are shown in the graph.

Ionizing radiation usually causes cell cycle disruption. To investigate cell cycle redistribution after a carbon ion beam (at the center of SOBP with 50 keV/µm of average LET) alone or in combination with 25 µM of cisplatin (CDDP), the cell cycle analyses of MESO1 cells were performed by FACS Callibur. As shown in Figure 8, carbon ion beam irradiation combined with cisplatin inhibited cell cycle progression (sub-G1 arrest) and induced death (apoptosis/necrosis) of MESO-1 cells with greater efficacy that did carbon ion beams, or cisplatin alone. γH2AX foci formation in CD44+/CD26+ and CD44+/CD26+ cells after carbon-ion beam alone or in combination with cisplatin

**Figure 9:**
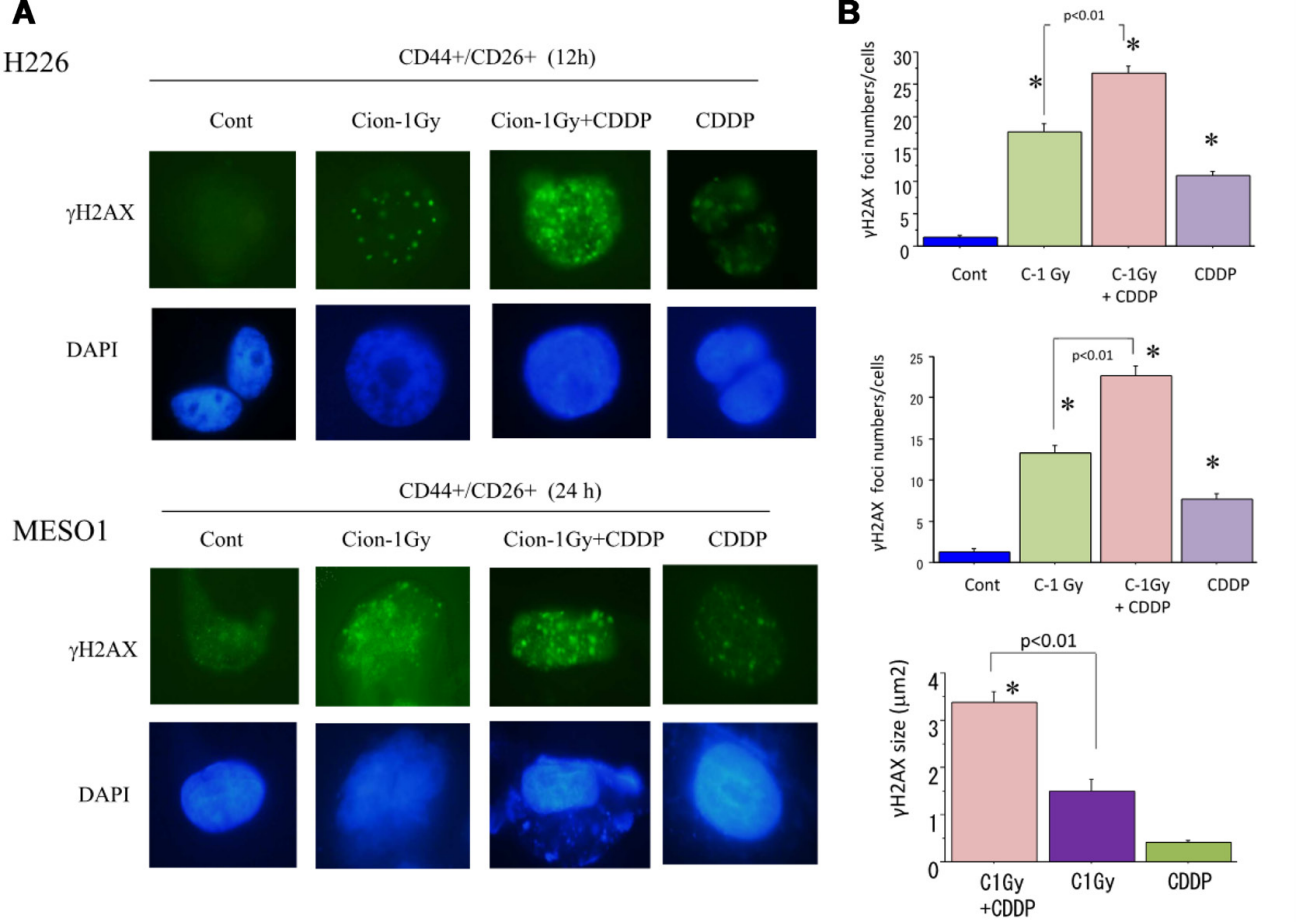
(**A**) Representative photos of nuclear γH2AX foci formation in CSCs (CD44+/CD26+) derived from H226 (12 h) and MESO1 (24 h) after a carbon ion beam alone or in combination with of CDDP (25 μM). CDDP was added prior to irradiation and treated for 12 h or 24 h. (**B**) Quantification of nuclear γH2AX foci formation in CSCs (CD44/CD26) derived from MESO1 cells at 24 h after a carbon ion beam alone or in combination with CDDP (25 μM). CDDP was added prior to irradiation and treated for 24 h. Data represent mean ± SD. ^*^p < 0.01 compared to γH2AX foci numbers in Control or sizes in carbon ion alone irradiated cells. All experiments were performed in triplicate (*n* = 3).

Ionizing radiation exposure can induce DNA double strand break (DSB), and γ-H2AX is a typical biomarker of DSB. To clarify DSB induction by carbon ion beam (at the center of SOBP with 50 keV/µm of average LET) alone or in combination with 25 µM of cisplatin, the number and the size of nuclear γH2AX foci formed in CSCs (CD44+/CD26+) delivered from MESO1 cells were examined. We found that a higher number of γH2AX foci were remained 24 h after treatment with carbon ion irradiation in combination with cisplatin, and the size of the γH2AX foci was significantly larger than that in cells treated by carbon ion irradiation alone (Figure 9).

## DISCUSSION

In the present study, the MESO1 and H226 cell viability 7 days after irradiation with carbon ion beam alone significantly decreased, and it was predominantly decreased after in combination with 25 µM cisplatin. We found that the RBE values of carbon ion irradiation at 13 and 73 keV/μm relative to X-rays in MESO1 (H226) cells were 1.19 (1.22) and 2.93 (2.82), respectively. Many factors including linear transfer energy (LET) influence the RBE values, and the data obtained in this study are partially in line with other reports where the range of RBE values was around 1.57–2.60 for carbon ion beams at 50–80 keV/µm [15, 16, 21]. Carbon ion beams combined with CDDP remarkably inhibited colony formation and decreased MESO1 cell survival. This is consistent with results of previous reports showing that CDDP is effective in killing mesothelioma cells [20, 22].

The present study indicated that CD44+/CD26+ cells have a significantly higher colony and spheroid formation capability compared to CD44–/CD26– cells derived from H226 cells, suggesting that CD44+/CD26+ cells possess CSC characteristics. Similarly, the CSC properties of CD44+/CD26+ cells in comparison with those of CD44–/CD26– cells derived from MESO1 cells have been confirmed based on their high spheroid formation capability. This is in line with previously reports that CD44+/CD26+ and/or CD44+/CD26+ are MM CSC markers [9, 10, 22, 23]. The proportion of CSC-like CD44+/CD26+ cells among MESO1 and H226 cells increased ten days after X-ray irradiation in a dose-dependent manner. In comparison no such response was induced by carbon ion irradiation. Interestingly, the proportion of CD44+/CD26+ cells was significantly increased by X-ray combined with cisplatin when compared to the combination of carbon ion beams and cisplatin combination or cisplatin alone. This change in the proportion of CD44+/CD26+ cells among H226 cells appeared to be similar with the results of our previous reports [17, 18]. Furthermore, the combination of carbon ion irradiation and cisplatin yielded significantly greater reduction of spheroid size than that achieved using X-rays alone, carbon ion beams alone, or the combination of X-rays and cisplatin, suggesting that cisplatin induced significant radiosensitization of MM CSCs to the carbon ion beam.

The FACS and caspase-Glo analysis indicated that carbon ion irradiation alone can significantly induce MM cell apoptosis and it was more predominantly enhanced by the combination of carbon ion beams and cisplatin in comparison with either carbon ion beams or cisplatin alone. This is partially in line with other reports whereby cisplatin was effective in inducing MM cell apoptosis [22]. Cell cycle analyses showed that combination treatment of carbon ion beams and cisplatin is more effective in inhibiting cell cycle progression (sub-G1 arrest) and inducing cell death (apoptosis/necrosis) of MESO1 cells compared with carbon ion irradiation alone. This is partly consistent with our previous report [18].

Increasing evidences have shown that CSCs are chemo-radioresistant in comparison with non-CSCs. The high DNA repair capability and slow cell cycle progression of CSCs allow them to protect themselves from many cellular stress such as radiation and anti-cancer drugs [24, 26]. Cisplatin, a typical DNA damaging drug has been reported to work as a radiosensitizer by inducing apoptosis and autophagy in MM cells [27, 28]. In this study, the data showed that remarkable increases in the expression of apoptosis-related BAX by carbon ion beam alone and autophagy-related Beclin 1 and ATG7 by combination treatment of carbon ion irradiation and cisplatin onr radioresistant CSCs derived from MESO1 cells. This finding indicates that cisplatin enhances CSC killing effect of carbon ion irradiation when used together. An increasing number of studies in the literature have determined that CSCs are appeared to be chemo-radioresistant [11, 25]. Based on the report that cisplatin has the potential to induce CSC differentiation in cancer cell lines [29], the beneficial effects of carbon ion irradiation combined with cisplatin in inducing apoptosis and autophagy are further supported in MM CSCs at the mRNA levels *in vitro*. However, further *in vitro* and *in vivo* studies are required to investigate genes at the protein level.

In this study, we found that the combination of carbon ion irradiation and cisplatin induced the formation of a higher number of larger-sized γH2AX foci, which is a marker of double strand breaks (DSBs), compared to either carbon ion beam or cisplatin alone treatment. This finding indicates that high-LET carbon ion beams combined with a DNA damaging anticancer drug such as cisplatin can results in irreparable clustered DSBs [31, 32]. These and other previously reported data support the fact that high-LET heavy ion irradiation can induce a higher number and large-sized γH2AX foci in radioresistant CSCs compared to conventional low LET X-ray irradiation [17, 18, 30].

Collectively, the combination treatment of carbon ion irradiation and cisplatin is superior to carbon ion irradiation alone because of the high effectiveness in killing putative MM CSCs by inducing apoptosis, autophagy, and irreparable DNA damage. In conclusion, cisplatin when combined with carbon ion beams, may have the potential to maximize the therapeutic effects of carbon ion radiotherapy for MM.

## MATERIALS AND METHODS

### Cell lines and reagents

Human mesothelioma cell lines, H226 and MESO1 were purchased from American Type Culture Collection (Manassas, VA). Unsorted cells were cultured in Dulbecco’s Modified Eagle’s medium (DMEM) supplemented with 10% heat-inactivated fetal bovine serum (Beit-HaEmek, Israel),100 unit/mL penicillin and 100 μg/mL streptomycin (Invitrogen) at 37°C with 5% CO2-in-air. The medium was changed every other day. CSCs and non-CSCs isolated from H226 and MESO1 cells were cultured with Cancer Stem Cell medium (Heidelberg. Germany). Cisplatin (CDDP) was purchased from Takara Bio Japan. The cisplatin solutions were diluted in PBS immediately before use. The concentration of CDDP mainly used in this study was 25 M based on previous reports [33, 34], which is suitable for evaluate its effects on mesothelioma *in vitro*.

### Colony and spheroid formation assays

Clonogenic survival assay was performed as described previously [15, 16]. In brief, the appropriate plating density was aimed at producing 20–40 surviving colonies in each T-25 flask. After incubation for 14 days, the colonies were fixed and stained with 0.3% methylene blue in ethanol, and colonies containing more than 50 cells were counted as survivors. At least three parallel samples were scored in three to five repetitions performed for each type of irradiation. Clonogenicity and spheroid formation ability assay for CD44+/CD26+ and CD44–/CD26– cells sorted from H226 cells and CD44–/CD26– and CD44+/CD26+ cells sorted from MESO1 cells were performed as described previously [15]. The data is presented as the percentage of the wells that contained spheres, and the average size using WinRoof 5.6 software (Mitani Corporation, Tokyo, Japan) after 1-week incubation.

### Irradiation

Cells were irradiated with carbon-ion beams (accelerated by the HIMAC). Briefly, the initial energy of the carbon-ion beams was 290 MeV/n, and the dose-averaged LET values of 13, 50 and 73 keV/μm at spread-out Bragg peak (SOBP), which were obtained from the initial energy of the 290 MeV per nucleon carbon ion beams were used. As a reference, cells were also irradiated with conventional 200 kVp X-ray (TITAN-320, GE Co.,USA).

### Cell viability assay

For the analysis of cell viability, a CellTiter-Glo luminescent cell viability assay (Promega) assay were used. The CellTiter-Glo^®^ Luminescent Cell Viability Assay is a homogeneous method to determine the number of viable cells in culture based on quantitation of the ATP present, which signals the presence of metabolically active cells. The CellTiter-Glo^®^ Assay is designed for use with multiwell plate formats, making it ideal for automated high-throughput screening (HTS), cell proliferation and cytotoxicity assays. The homogeneous assay procedure involves adding a single reagent (CellTiter-Glo^®^ Reagent) directly to cells cultured in serum-supplemented medium. Cell washing, removal of medium or multiple pipetting steps are not required.

### FACS analysis

FACS analysis for the cells irradiated with X-rays or carbon ion beams was performed with BD FACS Aria (Becton Dickinson, San Jose, CA, USA) as described previously [15] In brief, the cells were prepared and labeled with conjugated anti-human CD44-PE (Miltenyi Biotec), ESA-APC (Miltenyi Biotec), and CD24-FITC. Isotype matched immunoglobulin served as control. Cells were incubated for 20 min at each step and were washed with 2% FCS/PBS between steps. The percentage of CD44+, ESA+, and CD24+ present was assessed after correction for the percentage of cells reactive with an isotype control.

### Apoptosis analysis

The apoptosis was analyzed using Annexin-V/PI doubling staining flow cytometry assay with FACS using Annexin V-FITC Apoptosis Detection Kits, according to the commercial procedure available (R&D Systems, Minneapolis, MN USA). Briefly, after 24 h of irradiation cells were harvested by trypsinization, washed in PBS and labeled fluorescently for detection of apoptotic and necrotic cells by adding 100 μL of binding buffer and 1 μL of Annexin V-FITC to each sample. Samples were mixed gently and incubated at room temperature in the dark for 15 min. Immediately before analysis by flow cytometry (BD FACSCalibur Flow Cytometry System), 1 µL of propidium iodide (PI, 1 mg/mL; Cedarlane Laboratories, Hornby, Ontario, Canada) were added to each sample. A minimum of 10,000 cells within the gated region was analyzed.

### Cell cycle analysis

After harvesting and washing cells with PBS, fix in ice-cold 70% ethanol (ethanol in distilled water) while vortexing, then stained with propidium iodide (1 μg/mL, Sigma) in the presence of RNase A according to the manufacturer’s protocol, and then analyzed using a BD FACS Calibur flow cytometer (BD Biosciences). A minimum of 10,000 cells was counted for each sample, and data analysis was performed with CellQuest software [18].

### Real time RT PCR analysis of various gene expression related to apoptosis and, autophagy

RNA was purified using the Qiagen RNAeasy kit, including on-column DNAse treatment to remove genomic DNA. cDNA was prepared with the RT^2^ First Strand Kit (SABiosciences, Frederick, Maryland, USA). A PCR specific for apoptosis, autophagy related genes was performed (RT^2^ SYBR Green/ROX qPCR Master Mix; SABiosciences) in 96-well microtiter plates on a LightCycler^®^ 96 system (Roche, Basel, Switzerland). For data analysis, the ΔΔCt method was applied using the RT PCR software package and statistical analyses performed (*n* = 3). This package uses ΔΔ C_T_–based fold change calculations and the Student’s *t*-test to calculate two-tail, equal variance p-values. The fold changes were calculated using the equation 2^−ΔΔCt^. If fold change was greater than 1, the result was considered as fold-upregulation. If fold change was less than 1, the negative inverse of the result was considered as fold-downregulation [17].

### γH2AX immunofluorescence assay

γH2AX Immunofluorescence assay was performed as described previously [16]. In brief, cultured cells grown on plastic chamber slides (Lab-Tek. Nunc, USA) were fixed in 4% formaldehyde for 15 min at room temperature. Then the cells were permeabilized in 0.2% Triton X-100 and blocked with 10% goat serum, then incubated with mouse monoclonal anti-phospho-Histone H2AX(Ser139) (γH2AX) at 37°C in PBS with 10% goat serum and washed with PBS. The cells were incubated with the Alexa 488 anti rabbit secondary antibody at 37°C in PBS with 10% goat serum and washed in PBS. Cover glasses were mounted in ProLong^®^ Gold antifade reagent with DAPI (Invitrogen). Fluorescence images were captured using an Olympus DP70 fluorescence microscope for analysis. All treatment groups were then assessed for γH2AX foci via sequential imaging through each nucleus. A minimum of 100 cells in each treatment group were counted. Nuclear γH2AX foci size was estimated by ImageJ 1.45 software (NIH).

### Statistical analysis

One-way analysis of variance (ANOVA) and Bonferroni multiple comparison tests were used when mean differences between the groups were evaluated by StatView software (SAS Institute, Inc., Cary, NC). For all comparisons, *p* values less than 0.05 were defined as significant.

## Abbreviations

MM: malignant mesothelioma
CSC: cancer stem-like cell
HIMAC: heavy ion medical accelerator in Chiba
DMEM: dulbecco’s modified eagle’s medium
ANOVA: analysis of variance
SOBP: spread-out bragg peak
LET: linear transfer energy
RBE: relative biological effectiveness
